# Requirements for prevention reporting

**DOI:** 10.17886/RKI-GBE-2017-089

**Published:** 2017-08-30

**Authors:** Raimund Geene

**Affiliations:** Magdeburg-Stendal University of Applied Sciences, Department of Applied Human Sciences, Germany

## Abstract

Taking similar parliamentary reports and available expert opinions as a basis, we aim to establish specifications for the first prevention report due for release in 2019. We propose to develop a formative report based on intervention reporting under the guidance of an independent scientific board supported by an administrative office and guided by the overall aim of providing policy advice. By pooling available expertise in the field of prevention, we can promote an evidence-based focus and the development of indicators. The prevention report should contribute towards data harmonisation and the development of long-term monitoring structures. We strive to achieve arrangements with and anchor our work within federal, federal state and municipal structures.

## Target audiences of the prevention report

The prevention report constitutes the second pillar of Germany’s National Prevention Strategy and will be presented to the German Bundestag and Bundesrat in 2019. The report aims to provide legislators with the tools to comprehensively assess the development of health promotion and disease prevention measures and to identify those fields requiring action to update legislation. Book V of the German Social Code (SGB, § 20d (4)(4)) explicitly states that the prevention report should include recommendations to adapt spending guidelines for the services provided by health insurance funds. This aspect, in particular, is an unusual extension of the corporatist model of health insurance. As a rule, it assigns the framework to the legislative branch through the social code and, in individual delegation procedures (implementation decrees), it assigns it to the executive branch or subordinate legislators, such as the Joint Federal Committee (Gemeinsamer Bundesausschuss).

The prevention report will have the same rank as other scientific reports such as the Report on Children and Youth, the Family Report, the Elderly Report or the Report on Poverty and Wealth. These are regularly produced during each legislative period by expert committees, which are organised as separate agencies, and presented for discussion to the German Bundestag. In general, the commissions provide up-to-date summaries of research and use this as a basis to develop realistic, future-oriented options for action to be taken at the political and social level. Correspondingly, scientists sitting on the board of the commissions will then enlist the services of their agencies and/or request independent expert opinions provided by additional researchers and experts. The changes to the legislation concerning benefits for children and adolescents in accordance with book VIII of the SGB, which are currently being discussed as a grand solution (Große Lösung), are based on the proposals made by the 13th Report on Children and Youth and are just one example of the immediate political impacts of these reports.

The standards expected of the prevention report regarding scientific integrity and independence are thus high. To do justice to these demands, we can already refer to numerous qualified expert surveys (Table 1).

## Content of the prevention report

With the Preventive Health Care Act, legislators initiated a paradigm shift from disease prevention focused on behaviour to a settings approach. It would be desirable for the prevention report to follow this new focus and, in particular, to assess the strategies and effects of health promotion within determined settings.

Here it would be advisable to implement a set of modularly organised sub-opinions, for example, on measures of health promotion in child day care centres as they are currently being applied to evaluate Germany’s health target Grow up healthy [1]. Regarding the relevant health targets and sub-targets for child day care centres, a large number of data sources have been considered and their significance evaluated. Whilst this process revealed numerous gaps in the data and the analysis mostly relied on proxy variables, the number of activities did increase (output), even if this fact was not reflected to the same degree in the results (outcomes). Evaluation results show the need to improve quality orientation (also regarding the development and measurement of indicators) and continuous monitoring via a coordinating body.

Moreover, the evaluation results presented in this opinion include a recommendation to differentiate the health target Grow up healthy and develop Healthy kindergartens as a separate (sub-) target, which reflects the growing importance of this field. In this respect, the evaluation fulfils the demands made in the Preventive Health Care Act, according to which ‘the report should include recommendations to adapt the health targets developed by the forum gesundheitsziele.de in line with current demands or develop further targets’ [2].

## Processes of the prevention report

Overall, the prevention report should take a form that ensures that it can effectively contribute to the high demands of further developing Germany’s national prevention strategy, complement it as its second pillar, and, moreover, offer an adequate knowledge basis for subsequent hearings and debates in the German Bundestag and Bundesrat.

The report should therefore be produced by independent researchers, who may commission individual sections of the report for the purpose of the module on child day care centres’ health targets mentioned above. This could provide a core building block for the necessary pooling of expert knowledge on disease prevention, for example, by using a scientific approach to underpin an evidence-based method and pairing this with the development of indicators. Accordingly, the report should be process-focused (formative evaluation), include elements of intervention reporting and function as a basis for policy advice.

To implement this, the commission should set up independent structures, such as a separate agency and board, that are capable of providing an overview of current disease prevention activities, and can coordinate and focus efforts on specific issues. Through the report, the commission should contribute to data harmonisation and promote the creation of the structures required for long-term monitoring. This would require close collaboration between top-level federal agencies (Federal Centre for Health Education and the Robert Koch Institute), the federal states and municipalities.

## Figures and Tables

**Table 1:** Selected previous reports serving as a basis for the prevention report Own table

**Previous reports**
► Scientific studies of the BKK programme ‘Mehr Gesundheit für alle’ (Rosenbrock, Bellwinkel & Schröer 2004) [3] ► Report by the Advisory Council for Concerted Action in Health Care (Sachverständigenrats für die Konzentrierte Aktion im Gesundheitswesen, 2002, 2009) [4, 5] ► ‘Erkennen – Bewerten – Handeln’ (RKI & BZgA 2008) [6] ► Growing up healthy – KNP (BZgA 2012) [7] ► Evaluation of complex interventions (RKI 2012) [8] ► Health promotion in settings (BZgA & LVGs 2015) [9]
**and**
► Prevention reports (MDS & GKV-SV 2001ff.) [e.g. 10] ► Health report – Health in Germany (RKI 2015) [11]

BKK: Betriebskrankenkassen (company health insurance fund)

RKI: Robert Koch Institute

BZgA: Federal Centre for Health Education

KNP: Kooperation für nachhaltige Präventionsforschung (partnership for long-term preventive health care research)

LVGs: Landesvereinigungen für Gesundheit (state health associations)

MDS: Medizinischer Dienst des Spitzenverbandes Bund der Krankenkassen (medical service of the umbrella organisation of health insurance funds)

GKV-SV: National Association of Statutory Health Insurance Funds
